# The efficacy of hydro alcoholic extract of *Seidlitzia rosmarinus* on experimental zoonotic cutaneous leishmaniasis lesions in murine model

**Published:** 2014

**Authors:** Maryam Ahmadi, Abdolmajid Fata, Ali Khamesipour, Hasan Rakhshandeh, Akram Miramin Mohammadi, Ghodratollah Salehi, Hadi Monavari

**Affiliations:** 1 *Department of Parasitology and Mycology, Emam Reza Hospital, School of Medicine, Mashhad University of Medical Sciences. Mashhad, I. R. Iran*; 2 *Research Center for Skin Diseases & Cutaneous Leishmaniasis, School of Medicine, Mashhad University of Medical Sciences. Mashhad, I. R. Iran.*; 3 *Centre for Research and Training in Skin Diseases and Leprosy, Tehran University of Medical Sciences, Tehran, I. R. Iran.*; 4 *Department of Pharmacology and Medicinal plant Research Center, School of Medicine, Mashhad University of Medical Science, Mashhad, I. R. Iran.*

**Keywords:** *Cutaneous Leishmaniasis*, *Hydro-alcoholic extract*, *Leishmania major*, *Seidlitziarosmarinus*

## Abstract

**Objective:** Leishmaniasis is one of the most important parasitic infectious diseases in the world. Since last century, many efforts have been made to control and treat the disease, but appropriate vaccines, pesticides and medicines are not available or even eligible. The purpose of this study was to evaluate the effect of hydro-alcoholic extract of *Seidlitzia rosmarinus* on the lesions of experimental Cutaneous Leishmaniasis (CL) in Balb/c mice.

**Materials and Methods: **The population study was 60 Ballb/c mice which divided to 6 groups, all infected with *Leishmania major* [MRHO/75/IR]. Soon after the ulcer started to appear in the early stage, a dose of provided herbal extract with 5, 10 and 15% concentration applied on each lesion. The surface area of the lesions measured during an interval of 10 days. Direct Giemsa stained smears prepared two and four weeks after treatment.

**Results: **Increasing the mean size of the lesions was statistically significant compared to those in control group (p>0.001). Visceral Leishmaniasis (VL) developed in all of the mice including the control group that received Eucerine alone. Survival rate in group receiving 15% *S. rosmarinus* extracts showed significantly higher compared to mice in control group (p<0.001).

**Conclusion: **Hydro-alcoholic extracts of *S.rosmarinus* with concentrations below15% did not show a therapeutic effect on experimental CL ulcers of Balb/c mice. Further studies with higher concentrations or nano particles are recommended.

## Introduction

Alternative and herbal medicines have been approved by WHO (Fatahi et al., 2008 ▶). Leishmaniasis is a parasitic disease transmitted by sand flies. It is characterized by a spectrum of cutaneous, muco-cutaneous and visceral manifestations (WHO, 2006 ▶).

Cutaneous Leishmaniasis (CL) represents a common health problem and standard treatments are often ineffective or yield poor cosmetic results (Berman, 2003 ▶). The classic treatment is administration of pentavalent antimonials. The disadvantages of the antimonials are their expected side effects, invasive method and painful route of administration (Beheshti et al., 2007 ▶).

Traditional treatment of CL is a common habit of natives in many endemic areas (Weigel and Armijos, 2003 ▶; Kurban, 2000 ▶). Natural extract of different plants such as *Euphorbia* spp., *Gossypium herbacium*, and *Berberis vulgaris* are experienced by some investigators (Fata and Elahi, 1993 ▶). Although many therapeutic modalities have been suggested, but still there exist many problems associated with such methods of treatment (Nilforoushzadeh and Shirani-Bidabadi, 2008 ▶).

*Seidlitzia rosmarinus* with common name Glasswort is a perennial shrub, belongs to *Chenopodiaceae* family (Hedge et al., 1997 ▶). This plant grows on salty soils with high saline water tables as well as dry deserts. There are some reports that indicate this plant has medicinal properties and is used in dermatology medicine (Parsa, 1960 ▶). Ash remained after burning the leaves and stems has antiseptic and antibacterial efficacy (Mirheidar et al., 2000 ▶). Root tissues of *Seidlitzia rosmarinus* have a high capacity to absorb large amounts of soil alkaline materials such as Na+ and K+. It has also been reported that this plant has medicinal properties and is used for the treatment of some acnes (Parsa, 1960 ▶). The ash has also antiseptic and antibacterial properties (Mirheidar et al., 2000 ▶)

In ancient times, Iranian used to wash their clothing and even their bodies by Glasswort ash as detergent or soap (Hadi, 2009 ▶). In India, it has been used to exclude helminthes and because of burning efficacy, it has been rubbed on chronic lesions to eliminate keloid scars (Hadi, 2009 ▶ ).

Natives of Khorasan province traditionally use S*. rosmarinus* for treatment of CL lesions. Regarding limited previous experiments on possible efficacy of this plant against CL and to evaluate the efficacy of herbal extracts of *S. rosmarinus on *systemic Glucantime, this study was undertaken over a 12 months period in Department of Parasitology & Mycology, School of Medicine, Mashhad University of Medical Sciences and Centre for Research and Training in Skin Diseases and Leprosy, Tehran University of Medical Sciences.

## Materials and Methods

**Animals and groups**

Standard L*. major* (MHOM/ 64/IR/ER75) obtained from Pasture Institute Tehran, Iran. Promastigotes cultured in cRPMI1640 medium and incubated at 27°C.A total of 60 female Balb/c mice aging 4–6 weeks old were obtained from Pasture Institute. Each mouse inoculated subcutaneously by 0.5 ml liquid phase culture containing at least 4.8 l0^6 ^viable stationary-phase promastigotes. After 4 weeks, nodules and ulcers appeared on 54 inoculated mice. Then they were divided in randomized order into six equal groups. The rest uninfected mice excluded from the study. Four groups (36 mice) used in the experimental conditions and the others as controls.

**Plant and extract**

The plant, *S. rosmarinus,* (an isolate of Ghuchan district, Northeastern of Iran), obtained and diagnosed in Frdowsi University Herbarium (Herbarium No.2010, by Dr. Garivani). Then the stems and leaves of* S. rosmarinus *washed, dried and extracted by Soxhlet apparatus by ethanol 70% solvent in the Department of Pharmacology, School of Medicine, Mashhad University of Medical Sceinces. Mashhad, Iran.

**Experimental protocol**

About 0.5mm^3^of different concentrations (5, 10& 15%) of eucerine based hydro- alcoholic extract of *S. rosmarinus *applied on CL lesions of 3 groups of mice. The ointments rubbed topically two times daily for 30 days. Another group was treated by intraperitoneal injection of Glucantime (0.02 ml/g) once daily for 30 days. The control group A, received pure eucerine alone and the control group B remained without any treatment. Before using extract, both diameters of the lesions measured by Kulis Vernier. At the end of each 10 days interval, measurement repeated. Direct Giemsa stained smear was prepared from the lesions of experiment and control groups at second and fourth weeks post treatment. All groups of mice followed up for one month. 

**Statistical analysis**

All results were expressed as meanSD. The results were analyzed by Generalized Estimating Equations (GEE) and Marginal modeling. ANOVA and LSD tests were used to examine the changes. A probability level of *P*< 0.001 was statistically considered significant. Data were analyzed by SPSS software, version19.00.

## Results

**Figure 1:**
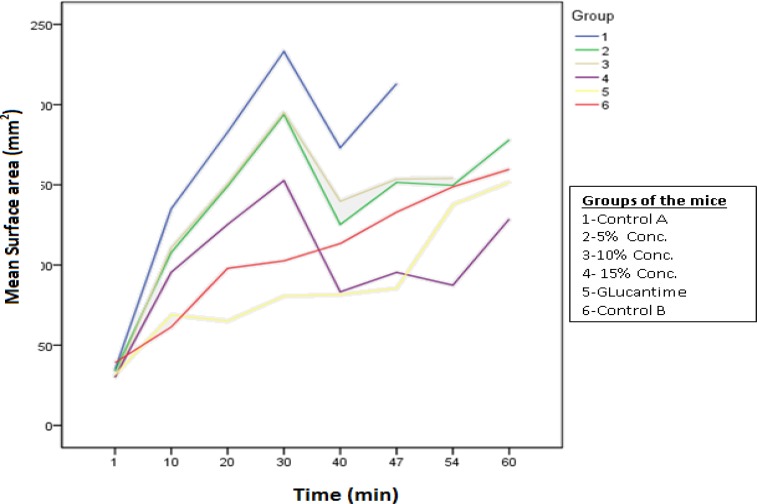
Progress of lesion size in inoculated Balb/c mice by *L.major* according to time duration

The results showed that at the end of treatment period, diameter of CL lesions increased in all 6 experimental and control groups. Statistically significant increase in the size of ulcers observed in 5% and 10% concentrations and in the placebo control group (*P*< 0.001). Treatments could not reduce the diameter or caused small lesions to disappear completely. These changes are shown in Table 2 and Figure 1.

Direct Giemsa stained smears prepared from the lesions of experimental mice showed positive for Leishman bodies. Two and four weeks after treatment, secondary bacterial infection of the lesions confirmed by observing bacteria and polymorphonuclear cells (Neutrophils) on stained smears obtained from treated mice. After discontinuation of treatment, a remarkable reduction of lesions’ size observed in all experimental groups except Glucantime (Figure 1).

To investigate the efficacy of different routes of treatment on the size of CL lesions by time duration regarding repeated measurements, a Marginal Model was used. The Model parameters are estimated by Generalized Estimating Equation (GEE), (Table 2 and Figure. 1). The results indicated that Glucantime was significantly more efficient to reduce the ulcer size in comparison with *S.rosmarinus* hydro-alcoholic extract regarding time duration. In this study the mice that have not received any treatment was regarded as reference group for statistical analysis.

Overall, the results showed a statistically significant increase in the lesion size of treated mice compared with reference group except for treated group by 15%extract and Glucantime group. Most significant increase of the lesion size observed in the mice (control A), that received Eucerine alone compared with reference group (P0.001).

**Table 2:** The results of Marginal modeling for comparing efficacy of different treatment on lesion size regarding time duration.

**Hypothesis Test**	**95% Wald Confidence Interval**		**B**	**Parameter**
**P-value**	**Upper**	**Lower**		
0.001	90.918	49.446	70.18±10.58	**[Eucerine ]**
0.014	58.584	6.591	32.58±13.26	**[5% extract]**
0.001	67.503	21.260	44.38±11.8	**[10%extract]**
0.842	39.277	-32.045	3.6±18.2	**[15%extract]**
0.105	3.856	-40.996	-18.57±11.45	**Gelucantime**

One month after the treatment period, the size of ulcers increased in the experimental and control groups which were still alive. Direct Giemsa stained smears obtained from 

live mice of all groups on second and fourth weeks post treatment showed positive result for Leishmania without any decrease in frequency of the Parasite.

## Discussion

Nowadays, efforts are continued to discover an effective route of treatment to cure CL with minimal side effects.

At the moment Glucantime, as an antimonial compound, is administered for all forms of Leishmaniasis. However Glucantime is used as a standard medicine, but it has many side effects. Therapeutic effect and adverse drug reaction (ADR) of Glucantime has been studied before (Beheshti et al., 2007 ▶). Erythema, edema, local pruritus and urticaria have been reported as ADR. Other rare untoward effects were local swelling, nausea and vomiting, diffuse erythema and shock. In the intramuscular group, the side effects were less in comparison to the intralesional group and they were urticaria, muscles pain, diffuse erythema, headache, edema or wound in the injection site, chills and fever (Beheshti et al., 2007 ▶). This means that there are still many problems in treatment of CL even by standard protocols.

Since ancient times herbal medicine has occupied a big space in the life of man (Nakhaei et al., 2010 ▶). Traditional treatment of CL is a common habit of natives in many endemic areas (Beheshti et al., 2007 ▶; Kurban, 2000 ▶). Natural extract of different plants such as*Euphorbia myrsinitis and Gossypium herbacium *are commonly used by patients in rural areas of Khorasan. Fata and Jafari in two different studies evaluated the efficacy of *Euphorbia myrsinitis* extracts (Fata and Elahi, 1993 ▶; Jaafari et al., 2006 ▶). In another study different concentrations of ethanol extract of the stem, leaves and root of *Berberis vulgaris*, were used topically on experimental CL lesions of Balb/c mice. The result showed a statistically significant decrease of ulcer size in experimental mice (Fata et al., 2006 ▶). Hydro-alcoholic extracts of* Achillea millefolium* and*Thymus vulgaris *experimentally used in another study. The results were suggested that the hydro-alcoholic extracts of the mentioned plant were significantly more effective in reduction of ulcer size as compared with Glucantime (Nilforoushzadeh and Shirani-Bidabadi, 2008 ▶). The effect of various concentrations of *Artemisia* essence in Balb/c mice was studied by Doroodgar et al. In that study the cutaneous lesions of the mice inoculated by *Leishmania major* enlarged following application of higher concentration of the *Artemisia* essence. Consequently, not only the lesions did not heal, but their size increased. In addition, parasitologic examination also remained positive (Doroodgar et al,. 2008 ▶). The result of latter study corresponds with the results of present experience. The same results also obtained by application of* Rubia tinctorum* extract on cutaneous leishmaniasis in Balb/c mice (Fatahi et al., 2008 ▶).* S. rosmarinus* has been traditionally used in Mashhad and its suburb regions for treatment of CL. Regarding rare available data about the possible efficacy of this plant against leishmaniasis, the efficacy of herbal extracts of *S. rosmarinus* against cutaneous leishmaniasis in Balb/c mice was examined in this study.

The natives in Khorasan Province use pure dried leaves’ powder of *S .rosmarinus* on their CL lesions. Therefore, alcoholic extract of stem and leaves, which is almost similar to the pure powder, used in this study. 

Using different concentrations of alcoholic extract showed different results (Fig.1). In this study, Eucerine was used as a base for the extracts, however, the results could be different if we had used Vaseline or Lanoline as a base for transdermal delivery of herbal extract (Fata and Elahi, 1993 ▶). A statistically significant increase of ulcer size observed in Balb/c mice which received Eucerine alone (control group A) comparing other groups, which approves the latter suggestion. The route of administration of drug is also important. In present study we used the ointments topically, but the results might be different if we could administer the extracts by intralesional route. In one study, successful treatment of oriental sore was reported by using intralesional berberine sulphate (Sabir et al., 1971 ▶).

Recent studies have shown that Nano particles of anti leishmanial drugs are highly effective to treat CL. The important advantages of such drugs are low needed dosage and minimum adverse reactions (Tafaghodi et al., 2010 ▶; Tafaghodi et al., 2011 ▶; Jaafari et al., 2009 ▶). Hydro-alcoholic extract of *S.rosmarinus* ( with concentrations below15%) did not show any therapeutic effect on experimental CL ulcers of Balb/c mice; but there is no statistically significant increase of ulcer size of treated mice by hydro-alcoholic extract of 15% *S. rosmarinus* and Glucantime. The higher concentration or dosage may have better therapeutic efficacy. Administration of Nanoparticles of herbal extracts should be considered. However, in order to reach such a strong conclusion, further studies are recommended.
